# The Synergy between Organ-on-a-Chip and Artificial Intelligence for the Study of NAFLD: From Basic Science to Clinical Research

**DOI:** 10.3390/biomedicines9030248

**Published:** 2021-03-02

**Authors:** Francesco De Chiara, Ainhoa Ferret-Miñana, Javier Ramón-Azcón

**Affiliations:** 1 Biosensors for Bioengineering Group, Institute for Bioengineering of Catalonia (IBEC), The Barcelona Institute of Science and Technology (BIST), Baldiri I Reixac 10–12, 08028 Barcelona, Spain; aferret@ibecbarcelona.eu (A.F.-M.); jramon@ibecbarcelona.eu (J.R.-A.); 2 ICREA-Institució Catalana de Recerca i Estudis Avançats, 08010 Barcelona, Spain

**Keywords:** NAFLD, extra-hepatic outcome, organ-on-a-chip, artificial intelligence

## Abstract

Non-alcoholic fatty liver affects about 25% of global adult population. On the long-term, it is associated with extra-hepatic compliances, multiorgan failure, and death. Various invasive and non-invasive methods are employed for its diagnosis such as liver biopsies, CT scan, MRI, and numerous scoring systems. However, the lack of accuracy and reproducibility represents one of the biggest limitations of evaluating the effectiveness of drug candidates in clinical trials. Organ-on-chips (OOC) are emerging as a cost-effective tool to reproduce in vitro the main NAFLD’s pathogenic features for drug screening purposes. Those platforms have reached a high degree of complexity that generate an unprecedented amount of both structured and unstructured data that outpaced our capacity to analyze the results. The addition of artificial intelligence (AI) layer for data analysis and interpretation enables those platforms to reach their full potential. Furthermore, the use of them do not require any ethic and legal regulation. In this review, we discuss the synergy between OOC and AI as one of the most promising ways to unveil potential therapeutic targets as well as the complex mechanism(s) underlying NAFLD.

## 1. Introduction

**Figure 1:**
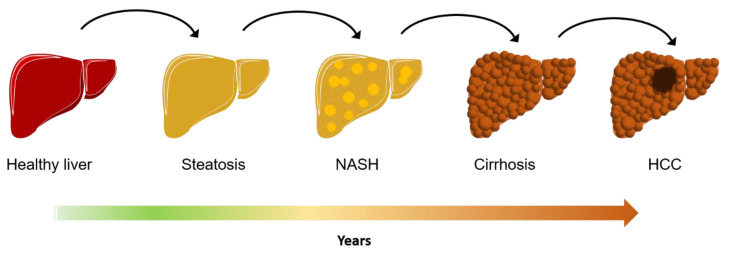
Representation of natural progression of Non-alcoholic fatty liver disease.

Non-alcoholic fatty liver disease (NAFLD) is defined as a continuum of abnormalities caused by lipid accumulation within the liver defined as hepatic steatosis. Fatty liver is considered as the liver manifestation of metabolic syndrome when is accompanied by simultaneously presence of three or more of the following features: high triglycerides, hypertension, visceral obesity, insulin resistance and high cholesterol, in the absence or reduced alcohol intake [1]. It ranges from non-alcoholic fatty liver (NAFL), characterized by > 5% of steatotic hepatocytes, to its more advanced and aggressive form known as non-alcoholic steatohepatitis (NASH). This latter form is characterized by extended liver inflammation and often accompanied by fibrosis which may progress to cirrhosis and hepatocellular carcinoma (HCC) (Figure 1). The strongest predictor of NAFLD/NASH is central or visceral obesity, rather than general obesity, while the presence of advanced fibrosis is the strongest predictor of mortality in those patients [2,3].

The global prevalence of NAFLD has been estimated around 25.24% with highest peak of 30.45% in South America, whereas NASH ranges from 3 to 5% globally. These numbers increase when other factors are considered, for example, the estimated prevalence of NAFLD and NASH in patients with type 2 diabetes mellitus rise up to 21.51% and 43.63%, respectively, while 51.34% and 81.83% in people affected by obesity [4]. The gold standard for the diagnosis of NAFLD/NASH is the liver biopsy although many non-invasive methods—ultrasonography, computed tomography scan and magnetic resonance imaging/spectroscopy—and various scores—NAS score, FIB-4, fatty liver index and NAFLD fibrosis score—are considered valid alternatives [5,6,7,8]. However, this potpourri of techniques, the intra- and inter-variability of pathologists in liver biopsies evaluation [9], and the non-standardized site location of biopsies [10] interfere with the inclusion of patients in clinical trials, but most important, compromise the possibility to assess the efficacy of treatments in clinical trials.

In the last 10 years, many technologies such as tissue engineering, sensing, and microfluidics are converging to build more sophisticated organ(s)-on-a-chip (OOC). The aim is to reproduce in vitro the physiological conditions for drug screening purposes in order to increase the success rate of clinical trials [11]. This system ranges from multicellular to multiorgan set-up in both healthy and diseased disposition, where the responses to perturbation/treatment can be monitored in real-time regardless from the mechanisms of action. The OOCs are changing the translational science paradigm being an extremely cost-effective surrogate to the animal testing with a far higher predictive power and shorter time of response. The impact of these tools is the ability to assess at same time the efficacy and toxicity of new treatments giving important insights about their biological activity. Those platforms are designed to deeper investigate the mechanism(s) of action of a specific disease, its early detection, as well as the effectiveness of a drug and its potential side effects. The OOCs are reaching a level of complexity comparable with real organs allowing the early identification of crucial features of NAFLD to intervene efficiently. The new OOCs 2.0 are high-throughput devices where mini-tissues in three-dimensional (3D) are subjected to a microfluidic regimen and integrated with a sensor platform capable of collecting massive amount of data in real time [12,13,14]. Just to give an idea, over 200 Gigabytes of data can be generated by a single time-lapse experiment of 72 h where three images of 10,000 cells (one brightfield plus two fluorescent bands) for six timepoints/hours at two Megabytes/each are taken. Another example is the over millions of datapoints that can be easily collected from a 384 wells microfluidic platform in real-time [15]. Unfortunately, at moment the data generated by these platforms have far outpaced our capacity to process and analyze them creating an important analytic bottleneck.

One of the most promising approaches is to employ machine learning and artificial intelligence to metabolize these unprecedent amount of data. Although traditional machine learning offers advanced data processing capabilities, the advent of its most important component, the deep learning, made possible to analyze massive unstructured data such as images, drug-target interactions and computational biology [16,17,18,19]. These AI-based techniques employ algorithms that learn without direct programming overcoming the limitation of human recognition boosting human knowledge in various fields.

In this review we will discuss: (i) the implication of other organs in the progression of the disease; (ii) how the recent advances in OOCs field might improve the knowledge on early onset of NAFLD; (iii) how the artificial intelligence could help in tasks such as classifying known NAFLD-associated features while identifying new ones.

## 2. Extra-Hepatic Outcomes: NAFLD as Multiorgan Disease

**Figure 2:**
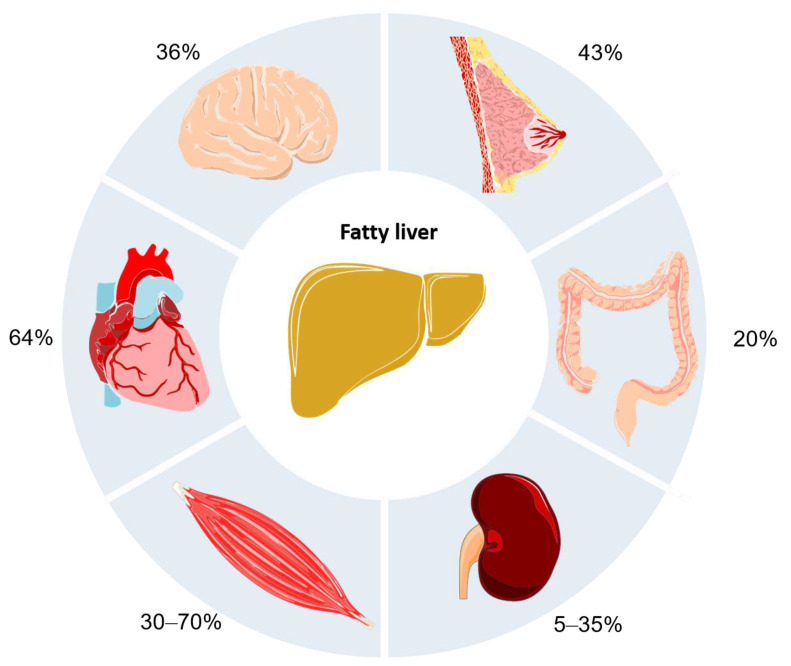
Incidence of extra-hepatic complication in non-alcoholic fatty liver disease.

In the past years, various lines of investigation have proved that NAFLD extends beyond the liver paving the way to multiorgan failure, and eventually death (Figure 2).

In the meta-analysis of Targher et al. [20], 64% of patients with NAFLD are at risk of cardiovascular disease (CVD), which represents the first cause of death. The outcomes range from non-fatal to fatal CVD complications such as angina, stroke, and myocardial infarction [21]. Obesity and other metabolic disorders like insulin resistance, atherogenic dyslipidemia, increased uric acid, reduced vitamin D, and impaired fibrinolysis are common risk factors of NAFLD and CVD [22].

Following CVD, the most frequent cause of death among NAFLD patients are the extra-hepatic malignancies, where colorectal cancer in males and breast cancer in females are the most prevalent types [23]. Specifically, Mantovani et al. [24] showed that in male patients with NAFLD, the prevalence of colorectal adenomas is 20.4% as opposed to 15.8% in those without NAFLD whilst the prevalence of colorectal cancer is 2.4% for NAFLD vs 1.97% without NAFLD [25]. On the other hand, NAFLD has been concurrently found associated with breast cancer in 45.2% of the female cohort vs the 16.4% of the controls [26]. Other evidence confirmed that visceral obesity, metabolic syndrome, and insulin resistance contribute to colorectal cancer progression [27], while disturbances in insulin metabolism, hormonal imbalance and inflammation are associated to higher risk of breast cancer [28].

Sarcopenia—referred as progressive and generalized loss of skeletal muscle mass and strength—is one more extra-hepatic complication that recently has been linked to NAFLD [29]. Between 30–70% of NAFLD patients with cirrhosis suffer sarcopenia [30], which increase the risk of worse outcomes in post-hepatic transplanted patients. Those patients have higher rates of mortality compared to non-sarcopenic patients [31]. Hence, sarcopenia is listed as exclusion criteria for a liver transplant [32].

Kidneys can be also affected by NAFLD. The decreased glomerular filtration rate and proteinuria, specific for chronic kidney disease (CKD), have been found in 20–55% of patients with NAFLD in contrast to 5–35% in patients without NAFLD. Moreover, there is a strong correlation between the stage of NAFLD and the severity of CKD [32]. Last evidence links the increased release of proinflammatory and prothrombotic molecules due to NAFLD to the promotion of vascular and renal injury [33].

The brain is also non-exempted from NAFLD-induced damage. Studies have associated NAFLD and cerebrovascular events (CVE), although data are frequently inconclusive. For example, El Azeem et al. observed CVE in 36% of NAFLD patients [34]. The associated complications were the asymptomatic brain lesions, alterations in cerebral perfusion and activity, cognitive impairment, brain aging, and increased risk of both ischemic and hemorrhagic stroke [35]. NAFLD and cerebrovascular accidents share some risk factors such as type II diabetes and obesity mainly related to metabolic syndrome [36]. Furthermore, recent findings indicate that the chronic inflammatory environment and ammonia generated by NAFLD might lead to microglia activation causing micro and macrovascular damage in the brain [37,38,39].

Other minor complications linked to NAFLD are skin-related (such as psoriasis), obstructive sleep apnea, osteoporosis, hormonal dysregulation (as polycystic ovary), male sexual disfunction, hypothyroidism, periodontitis, urolithiasis, etc. [40].

In NAFLD, multiple pathways are concurrently dysregulated. The liver homeostasis is chronically compromised by low level of inflammation and lipid accumulation. However, how this propagates to the other organs or vice versa the failure of other organs exacerbates the liver damage is still unknown. Further work is still urgently needed to elucidate the mechanism of interorgan cross talk in order to develop the right pharmaceutical target(s) or identify the right timing for treatment.

## 3. Organ-on-a-Chip 2.0

The organ-on-a-chip (OOC) revolutionized the way how the drug screening process was done, mimicking and miniaturizing the physiologic environment of almost any organ. In the last five years, these platforms have become more sophisticated thanks to a multidisciplinary approach. Three are the critical components of those platforms: the 3D tissue, the microfluidic, and the integrated sensing system.

**Figure 3:**
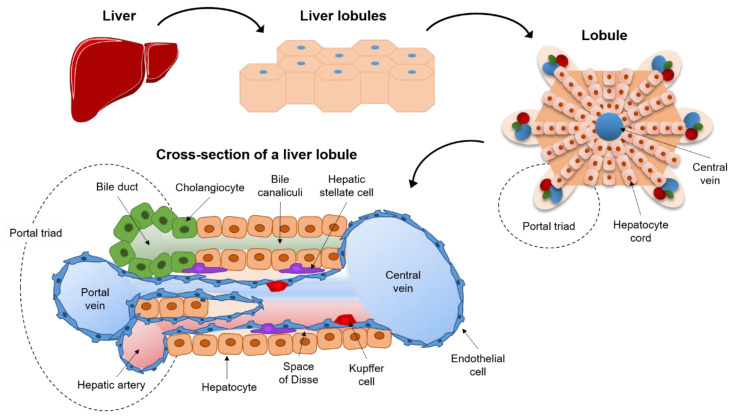
Schematic representation of liver lobule. The liver is composed of hexagonal functional unit known as lobule homogeneous throughout all the liver. Hepatic stellate cells give structural support to the endothelial cells which regulate the exchange with blood. Kupffer cells are the macrophage of the liver and responsible for the initial immune response in case of an insult. The hepatocytes represent the metabolic factory of the liver and organized according to their function. This anatomical configuration is the main challenge of tissue engineering.

The first of them is the three-dimensional structure obtained by mixing cells with a biomaterial in a pre-specified structure. In vivo, the liver is structured in hexagonal functional units defined lobules of 1–2 mm in size. At center of each lobule there is a large vein called “central vein” that drain blood from the small vessels (capillary) (Figure 3). At six corners, the lobules are delimited by the portal triad composed of bile duct, portal vein and hepatic artery. The cells placed between these two structures are organized according to their function. They are divided in non-parenchymal cells, composed by Kupffer cells (KC), liver sinusoidal endothelial cells (LSEC), and hepatic stellate cells (HSC) and parenchymal cells, hepatocytes, that constitute roughly the 20% and 80% of the liver mass, respectively. While the functions of non-parenchymal cells span from maintaining the structural organization of the liver (HSC) to regulate exchange with blood (LSEC) and immune response (KC), the function of parenchymal (hepatocytes) is to perform most of the metabolic jobs such as decomposition and synthesis of sugars and fats, ammonia removal and synthesis of bile acids. The place where the hepatocytes are located between the portal triad and central vein is extremely important for the body and it is known as zonation [41]. The environment of the cells closer to portal triad is rich in oxygen and glucose because the cells perform the processes that are more energetically demanding, such as glycolysis, bile acid production and xenobiotic metabolism. The mid-lobule hepatocytes are specialized in modulation of insulin growth factors and iron metabolism while the cells in the proximity of central vein are dedicated to gluconeogenesis, β-oxidation and cholesterol metabolism [41].

The cellular organization of the liver represents an essential factor for the hepatocytes’ optimal working condition but, at same time, the most significant challenge for the mimetic tissue engineering. In fact, when these cells are taken out from this configuration, they rapidly lose their polarization, capacity to replicate as well as some of the liver-specific functions. One of the first approach oriented towards the preservation of functionality of hepatocytes was made in the 1991 when Dunn et al. demonstrated that primary rat hepatocytes growth on collagen substrate were able to maintain polarity and release albumin, transferrin, and urea for two weeks. Once a second layer of collagen was added on top of these hepatocytes, those functions were prolonged for six weeks [42]. A different approach to prolong the hepatocytes’ phenotype, has been adopted by Suurmond et al. that cocultured hepatocytes, endothelial and Kupffer cells in 3D spheroids maintained in inter-connected honeycomb wells [43]. Interestingly, the presence of endothelial cells improves the function of hepatocytes—increase in urea albumin secretion—but not any other further improvement was induced by Kupffer cells. Conversely, the presence of the Kupffer cells determined an increase in lipid accumulation, and release of Tumor Necrosis Factor-α (TNFα) and Interleukin-6 (IL-6). These examples show that both the interaction with non-parenchymal cells and the presence of extracellular matrix are essential to recreate a useful in vitro model of NAFLD. Others crucial factors often neglected in many studies are the cell density and the contact with other cell types [44,45,46].

Recently, giant steps towards the replacements of extracellular matrix with synthetic biocompatible polymers have been made. These biopolymers with controlled diffusion properties mimic natural tissue stiffness, making it possible to keep hepatocytes’ phenotype and functions for a longer time. The most studied materials for tissue engineering are based on natural origins like gelatin, alginate, and cellulose [47,48,49] or synthetic materials such as poly L-lactic acid, poly (lactic-co-glycolic acid) and polycaprolactone [50,51,52]. Many variables, under the operator’s control, concur in the design and fabrication of these three-dimensional biopolymers. Porosity and stiffness are the essential variables to build a functional 3D structure compatible with cell viability and functionality [47]. The optimal pore size allows the cells to attach, interact, and form aggregates [53,54,55]. On the other hand, the scaffold must prevent the cells from escaping as well as constitute some sort of protection against the host’s immune system in case of implantation. Stiffness instead affects cell adhesion, motility, and polarization (referred to as the cell’s ability to differentiate in space and function). Generally, a healthy human liver has a stiffness that ranges from 3 to 8 kPa which can increase about 30% when fibrosis and fibrosis-related processes are in place [56]. Overall, rigid materials, with a stiffness that resemble the fibrotic state, enhance hepatocytes attachment, proliferation, mobility, and dedifferentiation [57,58]. It is biological plausible because of the intense cellular recruitment that takes place surrounding the damaged/fibrotic area where a high number of undifferentiated cells are needed to replace the dead ones. On the other hand, gene expression and the release of soluble markers of liver functionality, increase in hepatocytes encapsulated in soft substrates [48].

Microfluidic and shear stress have been shown to improve cell phenotype in vitro. An OOC is by definition a microfluidic cell culture device fabricated using microchip manufacturing techniques. These tools are provided with continuously perfused chambers where living cells are arranged to simulate tissue- and organ-level physiology. These devices produce tissue and organ functionality not possible with conventional 2D or 3D culture systems by recapitulating the body’s physicochemical microenvironments and vascular perfusion. Control of fluid flow in chips has proved enormously useful. Viscous forces dominate over inertial ones at small length scales, then the flow is laminar if the ‘microfluidic’ channel’s diameter is less than about one millimeter. Laminar flow allows the generation of physical and chemical gradients, which have been exploited for non-invasive study of directional cell migration [59,60,61], cardiac tissue formation [62], nerve axon outgrowth [63], and graded metabolic [64], differentiation [65], and neurotoxin [66] responses, as well as analysis of subcellular structure [59] and cell-cell junctional integrity [67]. Fluid shear stresses can be controlled independently of physical and chemical gradients by altering flow rates or channel dimensions [68,69] and separating cells from the flow path using a nano porous membrane [68] or micro-engineered posts that restrict cell passage [70]. Fluid-mechanical computational models can be applied to optimize microchannel geometry and enhance oxygen and nutrient delivery, thereby increasing cell survival and function [68].

**Figure 4:**
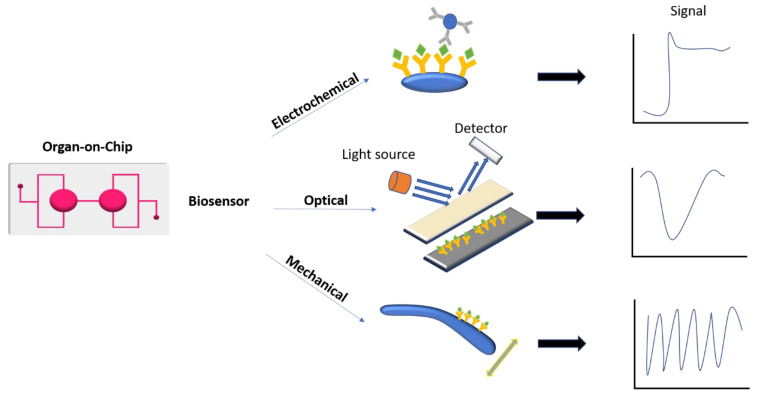
Examples of sensor platforms that can be integrated with organ-on-a-chip. Electrochemical biosensors measure the change of potential, current, conductance, or impedance induced by the immunoreaction. Optical biosensors determine the change in the optical properties of interaction between analyte and receptor. Mechanical biosensors assess the angular deflection due to molecular binding.

Accurate representation of in vivo organ physiology and drug pharmacokinetics can be achieved in OOC devices through precise control of cellular microenvironments; however, quantitative information extraction is equally essential. OOC integrated with new sensing technology allows easier intra- and extracellular measurements across different tissues. Biosensors are analytical devices consisting of a biological component (enzyme, receptor, oligonucleotide, cell, antibody, etc.) in intimate contact with a physical transducer that convert the biorecognition process into a measurable signal (electrical or optical) (Figure 4).

Because of its simplicity electrochemical transduction is the oldest and most common methods used in biosensors. They can determine the level of biomarkers by measuring the change of potential, current, conductance, or impedance caused by the immunoreaction. From all possible electrochemical transduction systems those based on recording amperometric signal generated after an enzymatic reaction have seen greatest development [71]. Amperometric biosensors are based on the measurement of the current generated by oxidation/reduction of redox species at the electrode surface, which is maintained at an appropriate electric potential. The current observed has a linear relationship with the concentration of the electroactive species. The electrode is usually constructed of platinum, gold, or carbon.

Optical transducers are based on various technologies involving optical phenomena, which are the result of an interaction of analyte with receptor. This group may be further subdivided according to the optical properties that have been applied in sensing (i.e., absorbance, reflectance, luminescence, fluorescence, refractive index, and surface plasmon resonance (SPR), and light scattering). The plasmon resonance is an evanescent electromagnetic field generated at the surface of a metal conductor (usually Ag or Au) when excited by the impact of light of an appropriate wavelength at a particular angle (α). Since distinct SPR prototypes (Biacore, IASys, etc.) have appeared in the market, a significant number of applications of this principle have been reported during the last years. Recently, localized surface plasmon resonance (LSPR) has been confirmed as a perfect candidate for OOC integration [72].

Microcantilever (MCL) translate molecular recognition of biomolecules into nanomechanical motion that is commonly coupled to an optical or piezo-resistive read-out detector system. Microcantilever sensors rely on their deflection to indicate sensing. Thus, molecular adsorption onto the sensing element shifts the resonance frequency and changes its surface forces (surface stress). Surface stress due to conformation change of proteins and other polymers has been a recent focus of MCL research. MCL that respond to conformational change-induced surface stress are promising transducers of chemical information and are ideal for developing microcantilever-based biosensors. (Figure 4).

OOCs can be employed to study the crosstalk between different cell populations of the same organ allowing further understanding of complex metabolic diseases. Current OOCs assays heavily rely on fluorescence microscopy and have mainly been used for specialized proof-of-concept studies, for example, in angiogenesis [73,74,75], in electrophysiology [76,77], and in pharmacological modulation of cell growth [76,78,79]. However, fluorescent labelling is a qualitative method and end-point assay losing all the real-time changes in metabolic behavior of the cells. There are few examples of OOCs where 3D functional tissues are integrated with an in-built sensor platform. Some lab-on-chip assays allow to measure parameters, such as oxygen concentration [80,81,82], pH level [83,84], and glucose consumption [85]. The use of antibodies add specificity to the sensors in detecting biomarkers, such as insulin or IL6 from complex biological media [71,86]. These biosensors are based on redox or enzymatic reactions that imply incubation and washing steps therefore limiting the value of the data acquired and not providing real-time information. Ideally, the next OOC platforms equipped with sophisticated built-in sensors would provide real-time data at cellular level.

## 4. Artificial Intelligence and NAFLD

Since 1970, when the potential of applying artificial intelligence technology in clinical setting was discussed, only few AI solutions were successfully employed. The main tasks performed were literature mining, automated experiments, and data collection [87,88,89,90]. For the following 20 years, there was a general loss of interest towards AI solutions in medicine known as “AI winter” that lasted until the end 90′s when “big data” became available. According to the website ClinicalTrials.gov, the ongoing clinical trials using big data and artificial intelligence are 3417 and 405, respectively.

**Figure 5:**
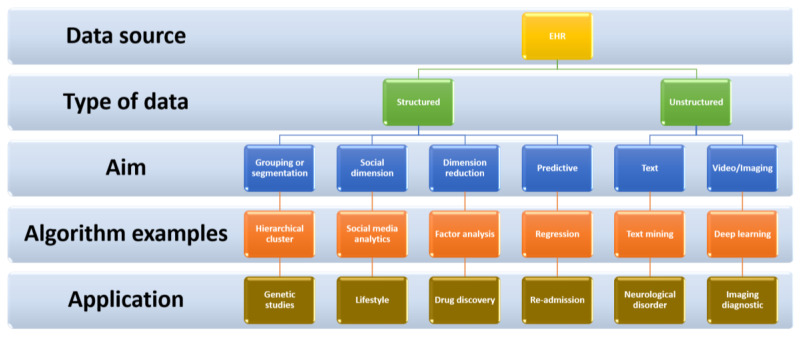
Electronic Health Records (EHR) workflow.

AI is the ability of a computer-based machine to apply a pre-determined set of rules (algorithm) to solve a specific task. By definition, these machines have the capacity to accurately predict a determined outcome or even take a decision that mitigates that outcome in a timely fashion. Both capabilities are prerogative of most human beings. To succeed in their task, these machines need to process a set of initial values (input) to generate a different set of values (output) thanks to a task-tailored algorithm. AI technology is a collection of physical (e.g., intelligent prostheses for handicapped people, robot performing surgeries) and virtual (e.g., predictive models that guide the clinicians’ decisions) applications. The Electronic Health Records (EHR) keep records of both practice and clinical management of patients during episodes of patient care and represents the primary reason of the tremendous upsurge of the virtual retrospective studies [91] (Figure 5).

**Figure 6:**
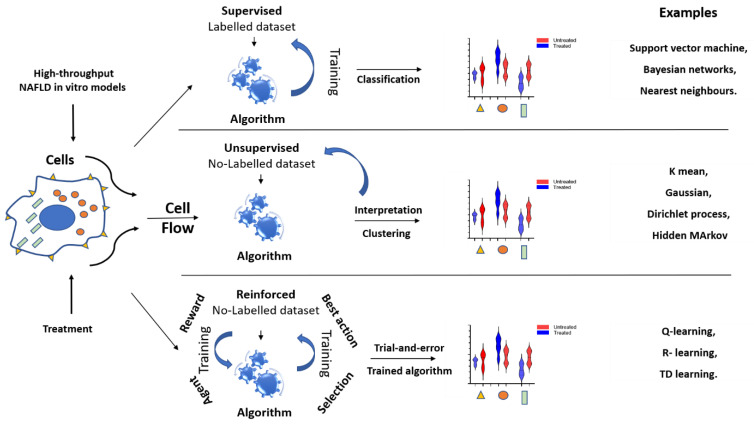
Supervised, Unsupervised, and reinforced learning applications for organ-on-a-chip.

Whilst computers can be programmed to perform better and more efficient tasks compared with humans, we are years far from AI tools capable to replace the latter in the medical practice, and more specifically, in the decision-making process. Machine learning, and its most powerful component deep learning, are crucial to make a machine able to acquire, analyze and interpret data in humanly fashion. The three most common approaches used are unsupervised, supervised, and reinforced learning [92]. The difference between the first two is the presence or not of a clean and labelled dataset to train the algorithm in order “to learn”. The limitation of these two approaches is the need of large dataset to train properly the algorithm, in case of supervised learning, and the low accuracy for the unsupervised. Differently, a reinforced algorithm interacts continuously with environment getting a feedback from it [93,94]. Every time it performs a task, it gets back an index, generally higher for the success and lower for the failure of the task. In that way, the algorithm modify itself to tend always to the higher index possible. However, this can lead to longer waiting time (Figure 6).

Nowadays, we are just mining massive amount of data to get insights about diseases. The next and closer step is to carry out predictive analysis to detect the early onset of the disease or at least identify its stronger risks factors. However, in medicine the main limitations are (i) the short follow-up of the patients—that reduce the predicting power of the models applied—and (ii) the inclusion during the analyses of potential hidden cofounding factors—that limit the matched-pair in clinical trial design causing a failure of the study. So far in NAFLD, only few papers have attempted to apply and implement the use of AI and machine learning mainly for its diagnosis. For example, Han Ma et al. investigated 11 machine learning (ML) techniques in a cross-sectional study of 2522 patients with NAFLD (assessed by ultrasonic examination), coming up with the conclusion that “users could focus only in five features—body weight, triglycerides, alanine aminotransferase, gamma-glutamyl transpeptidase, and levels of serum uric acid—that contribute to the NAFLD phenotype”. In this study, the Bayesian network model gave the best performance (the Bayesian network model consists in a graphical scheme where the nodes or features are connected each other according to causal relationship among them) [95]. A different approach has been used by Heinemann et al. where they attempt to automate the Kleiner score—ballooning, inflammation, steatosis, and fibrosis as main histopathological features—for the diagnosis of NASH for NAFLD/NASH models using a whole slide of liver rat and mouse stained by Masson’s trichrome. Four models of convolutional neural networks for image classification have been employed. Although it seems a very interesting approach, it is very difficult to apply to human [96]. First, this approach needs years of validation performed by expert pathologists to train the algorithm properly. Second, the high variability of the human evaluation of liver biopsies already in place using standard features. Third, the human biopsy is a tiny portion of tissue which most of the time is not representative of the whole organ.

Despite the advantages in applying AI for the diagnosis or treatment of patients, many challenges still exist in the quality and quantity of the collected data. First, the reduced number of samples affect the consistency and accuracy of the analysis. Second, the implementation of AI in medicine is faster than the ethical and legal regulation of the field. From this point of view, the biggest issues are data privacy, safety, and transparency [97].

## 5. In Vitro NAFLD Features Recognition: The Synergy between OOC and AI

The application of AI to the OOCs enables to analyze parameters that could not be considered so far for the study of NAFLD. Furthermore, their low in cost and automation propel them from engineering research into to any biomedical fields. With incredibly fast progress of OOCs in mimicking the complexity of native anatomical structures, many deep learning techniques developed for human can be applied. Deep learning is the biggest part of machine learning techniques and it is employed to recognizes pattern from the data using many levels of interpretation of unstructured data (images, text etc.). Its main limitation is the use of big training dataset that, in case of OOCs, is resolved using single cells images. For example, Guo et al. developed a high-throughput single cell lipid screening using an optofluidic time-stretch quantitative phase microscopy [98,99]. The combination of the quantitative phase imaging and machine learning allows the physical classification of cells (shape, area etc.) with high accuracy. This system can spot rare or even unknown events without a training dataset (unsupervised learning) and applicable to various fields. For example, it could be employed to study the kinetic of di- and triglyceride accumulation in spheroids of primary human hepatocytes trough imaging [100]. The rationale would be the understanding of fat trafficking inside the cells and its timing.

In another example, Blasi et al. demonstrated that it is possible to determine the cells’ DNA content, live or fixed, using brightfield and darkfield images obtained via imaging flow cytometry. Moreover, the cells’ mitotic phase can be also assessed [101]. The machine learning algorithm is trained against a dataset specific for the cell type or the interested feature. An interesting application would be the assessment of the in vitro regenerative capacity of hepatocytes according to their cell cycle phase. In fact, in patients with NAFLD, liver regeneration is compromised making these patients not optimal recipient for a transplanted organ [102]. One more study conducted by Chu et al. employed a machine learning based control system for microfluidic microencapsulation [103]. This system assesses via real-time the quality of the microencapsulation using spheroids images. This technology could be very useful to spot abnormal spheroids in a 3D printing high throughput platform therefore increasing the reproducibility for drug screening purposes.

These are only three examples of new technologies built for machine and deep learning techniques that might be applicable to identify the early pathological phenotypic changes of the cells or evaluate the efficacy of drugs candidates. On the other hand, many are the algorithm generated to analyze old data or applicable to old technologies. For example, Das et al. used light microscopic image acquisition from stained slides to extract 96 features to discriminate between Plasmodium-infected and non-infected erythrocytes [104]. The ability to discriminate the healthy or diseased status according to the shape would be extremely interesting in evaluating the change in shape in hepatic stellate cells strictly in contact with hepatocytes [105].

In another example, Mirsky et al. employed interferometric phase microscopy to develop a machine learning based classification method for sperm analyses and characterization [106]. The algorithm was able to extract 89 custom-designed features. This technology could be easily applicable to measure the degree of lysosomal permeabilization in hepatocytes upon treatment with saturated and unsaturated lipids [107]. This would give important insight about cell death. Ko et al. combines the nanofluidic technology with machine learning to discriminate between patients suffering of pancreatic cancer and healthy subject [108]. Although few human samples were analyzed, this study showed the potential of combining different technologies, in this case, nanofluidic, exosome screening and machine learning to better characterize patients in order to improve the success/failure ratio of clinical trial and/or treatment effectiveness.

## 6. Body on a Chip: An Exponential Growth in Complexity

The liver is the shield of the body protecting it from endogenous and exogenous substances. Once it fails in its function, many other organs are subjected to a continuous pathogenic signal that inexorably will lead to their failure, on a short time (acute) or a long time (chronic), if not neutralized. However, little information is available about the actors and their kinetic in pathogenesis of NAFLD.

Very few multiple organs on a chip have been developed in the last years to unveil the crosstalk between organs during the pathology. For example, Lee and Sung built a gut-liver on a chip to study the lipid accumulation as well as a potential treatment for reducing it [109]. Although the model is based on the use HepG2 (hepatoma cell line)—phenotypically and genotypically very different from healthy hepatocytes—they showed the importance of proinflammatory cytokine Tumor Necrosis Factor-α on the gut absorption permeability of the lipids. Interestingly, TNFα per se, did not exert any effect on the lipid’s accumulation in liver cells. On the same line, Ahluwalia et al. cocultured hepatic, endothelial, and different amount of adipose cells in an interconnected tissue models for the study of obesity and its lipid-related molecules and pro-inflammatory markers [110]. They showed the increase of pro-inflammatory markers such as Interleukin-6 and Monocyte chemoattractant protein-1 in endothelial and liver cells when connected to adipose tissue demonstrating the adipose tissue as an important source of inflammatory cytokine production.

These are only two examples of multiple organs interaction under the high load lipids intake regimen. However, a far more complex platform is necessary to identify both the players and the temporal role of them during the pathogenesis and progression of NAFLD. Real-time sensing system that targets concurrently multiple proteins and soluble factors and a computer-aided system to help in the analysis and interpretation would dramatically transform these platforms in the next few years. More important than a technological transition, it is the interaction of different fields such as biology, physiology, medicine, and engineering that will allow the full comprehension of the data generated by these platforms.

## 7. Conclusions

NAFLD is a multifactorial disease with unknown cause(s). Machine and deep learning are mainly used to improve the performance of OOCs. At the moment, the real job of these techniques is to reduce the dimensionality of the large number of variables or measurements to allow us to extrapolate useful information. The marriage between OOCs and artificial intelligence overcome the risk to apply AI-based application directly to human and therefore no need of ethical approval. In the next 10 years, artificial intelligence will turn these devices output in a real-time source of clinically relevant information improving medical decision. It is not too far to imagine a multi-organ on a chip completely controlled by algorithm that regulates the fluidic, the injection of treatments the measurement while correlates and interprets the outcome at same time. The combination of these two technologies will allow to analyze not only standard but also new features helping the pharmaceutical companies for screening of massive number of molecules in very short period. Furthermore, it will give a more exhaustive information compared with the traditional methods so far employed such as: discovering synergy between treatments, finding more effective concentrations, unveiling, and reducing potential side effect of drugs. From the patient’s point of view, the incorporation of these new OOCs could improve the stratification of recruited patients in clinical trials, saving cost and time, accelerating the development of precision medicine, and helping the clinicians in the diagnostic.

## Data Availability

Data sharing not applicable.
